# Palliative Radiation Therapy for Vertebral Metastases and Metastatic Cord Compression in Patients Treated With Anti-PD-1 Therapy

**DOI:** 10.3389/fonc.2019.00199

**Published:** 2019-03-29

**Authors:** Muhammad Mohsin Fareed, Luke R. G. Pike, Andrew Bang, Mai Anh Huynh, Allison Taylor, Alexander Spektor, Mark M. Awad, Patrick A. Ott, Monica Krishnan, Tracy A. Balboni, Jonathan D. Schoenfeld

**Affiliations:** ^1^Department of Radiation Oncology, Dana-Farber Cancer Institute, Brigham and Women's Hospital, Harvard Medical School, Boston, MA, United States; ^2^Department of Radiation Oncology, Princess Margaret Cancer Centre, Toronto, ON, United States; ^3^Department of Medical Oncology, Dana-Farber Cancer Institute, Harvard Medical School, Boston, MA, United States

**Keywords:** vertebral metastases, spinal cord compression, palliative radiation therapy, immune checkpoint blockade, PD-1 inhibitors

## Abstract

**Background:** There is increasing use of immune checkpoint blockade (ICB) across multiple cancer types, including in patients at risk for vertebral metastases and cord compression. These patients are often treated with palliative radiotherapy (PRT); however, data evaluating the combination of PRT and ICB in patients with vertebral metastases is limited. Furthermore, patients with cord compression are generally excluded from prospective clinical trials. Therefore, we retrospectively evaluated outcomes following PRT and PD-1 inhibition in patients with vertebral metastases.

**Methods:** We performed a retrospective chart review of 37 consecutive patients (total 57 lesions) treated with radiation for vertebral metastases who also received PD-1 inhibition. Patient, treatment and outcomes data were abstracted from the medical records.

**Results:** Histologies included non-small cell lung cancer (*n* = 21), renal cell carcinoma (*n* = 9) and melanoma (*n* = 7). Out of 57 lesions,18 involved >1 segments of the vertebral column. There were isolated lesions in thoracic (16), lumbar (9), cervical (6), and sacral (8) vertebrae. Presenting symptoms included pain (19), numbness (10), and weakness (3). Eleven patients were asymptomatic. Radiologic cord compression was present in 12, epidural extension in 28 and compression fracture in 14. Eleven patients underwent surgical decompression prior to the onset of RT. Median radiation dose was 24 Gy (range 8–30 Gy). Stereotactic radiation was delivered in 4 patients; 33 patients received conformal RT. 21 patients received PD-1 inhibition after RT, 9 before RT and 7 with RT. Seven patients received concurrent CTLA-4 inhibitors with anti-PD-1 therapy.

Treatment was in general well-tolerated. Toxicities included fatigue (6), transient pain flare (1), nausea/vomiting (1) and G1 skin changes (1). All patients reported some degree of pain relief. Numbness/weakness was improved in 6 of 13 patients with baseline symptoms (46%) and this was more likely in patients that received vertebral radiation after starting PD-1 inhibitors (71 vs. 17%, *p* = 0.04). Most patients (22 of 33 evaluable patients, 67%) had stability of irradiated lesions on subsequent follow up imaging performed at median of 30 days from RT, whereas 3 had a complete local response and 4 had a partial local response.

**Conclusions:** We demonstrate that PRT administered to vertebral metastases was well-tolerated and effective in patients treated with PD-1 inhibitors. There was an encouraging rate of pain reduction and neurological improvement.

## Introduction

With the addition of PD-1/CTLA-4 inhibitors to its armory, immunotherapy has changed the treatment landscape in oncology (1). The use of immunotherapy in combination with radiation is increasing across the spectrum of advanced disease, including those with disease metastatic to the vertebral column at risk for spinal cord compression. In these and other patients, the combination of PD-1 immunotherapy with ionizing radiation is of interest because of preclinical and clinical data suggesting that radiation may have the ability to support anti-tumor immunity via interferon mediated T-cell responses (2, 3). Multiple promising retrospective and prospective studies have been published over recent years demonstrating positive immune effects and clinical outcomes using a combination of targeted radiation and immune checkpoint blockade (2, 4–7). Perhaps most notably, the addition of the PD-L1 inhibitor durvalumab following chemoradiation as treatment for locally advanced non-small cell lung cancer (NSCLC) provided a significant progression-free and overall survival benefit to a degree not generally observed in the metastatic setting (8). In addition, PD-1 blockade may be hindered by disease burden and/or rapidly progressive disease (9, 10); this may represent a promising setting where radiation and immunotherapy can be combined to maximize effect as opposed to the alternative of patients coming off systemic therapy to receive palliative local therapy.

In patients with symptomatic vertebral metastases and metastatic cord compression, palliative radiation therapy plays an important role in symptom relief (11, 12). Thus, as the number of patients with vertebral metastases treated with PD-1 pathway inhibitors increases, evaluating the combination of these inhibitors with spine directed radiotherapy is of increasing importance. Despite the potential for overlapping toxicities, the combination of palliative radiation and PD-1 inhibitors has generally been well-tolerated in initial experiences (2, 13). However, patients with vertebral metastases and particularly metastatic cord compression is a unique population of patients often excluded from prospective clinical trials, where there are particular concerns related to tumor associated swelling and neurologic deficits. Furthermore, response rates and symptomatic improvement in patients treated with either palliative radiation or PD-1 pathway inhibitors are not well-defined. Therefore, we used our multi-institutional cohort of patients to evaluate outcomes specifically following PD-1 inhibition and palliative radiation delivered to patients with vertebral column metastases, focusing on toxicity as well as clinical and radiologic response.

## Materials and Methods

We performed a retrospective review of 37 consecutive patients with non-small cell lung carcinoma (NSCLC), metastatic melanoma and renal cell carcinoma (RCC) who received palliative radiation treatment for vertebral column metastases as well as PD-1 inhibitor therapy and / or CTLA-4 inhibition as per standard of care between August 2007 and September 2015. Subjects were identified from a comprehensive palliative care radiation oncology database including patients treated at 4 affiliated institutions under the approval of a single institutional review board as previously described (13). Indications for radiation therapy included painful bone metastases, sensory, motor deficits and/or spinal cord compression. All patients had both diagnostic imaging (generally with magnetic resonance imaging, *n* = 30), and CT guided radiation planning.

For our analysis, we reviewed clinical and radiation records using our Epic EMR system (Epic Systems, Verona, WI), as well as the ARIA OIS treatment planning system (Varian Medical Systems, Palo Alto, CA). Timing of radiation treatment relative to PD-1 inhibitor administration was calculated from the nearest day on which radiation was delivered relative to receipt of a PD-1 inhibitor. Analogous data was acquired for patients who received a CTLA-4 inhibitor.

Radiation therapy related adverse events were documented using Common Terminology Criteria for Adverse Events (CTCAE) version 4.0 (14). Response was judged per RECIST 1.1 criteria (15) using interval CT and/or MRI performed approximately 1 month (range 7–78 days) following the completion of radiation therapy. RECIST criteria were used for this study as opposed to immune response criteria (either irRC or iRECIST) to maximize the number of evaluable patients in this retrospective dataset and to facilitate comparison to other non-immunotherapy studies. Local response criteria were similarly defined: complete response (CR) was disappearance of the primary irradiated tumor; partial response (PR) was a decrease of 30% or more in the longest diameter of the primary irradiated tumor; progressive disease (PD) was an increase of 20% or more in the longest diameter of the primary irradiated tumor, and stable disease (SD) was recorded in patients whose tumors did not show either sufficient shrinkage to qualify for PR or a sufficient increase to qualify for PD.

We used descriptive statistics to report and summarize the patients' demographics, immunotherapy and radiation treatments, response to treatment and adverse effects of treatment. Median survival was calculated using the Kaplan-Meier method, and comparisons made using the log-rank test.

## Results

### Patient and Treatment Characteristics

We identified 37 patients treated with both PD-1 inhibitors and palliative spine radiation−21 with NSCLC, 9 with RCC and 7 with melanoma. Patient characteristics are shown in Table 1. The most common presenting symptom was pain (*n* = 19, 52%), followed by numbness or tingling (*n* = 10, 28%) and motor weakness (*n* = 3, 8%). Eleven patients (30%) were asymptomatic with concerning vertebral lesions incidentally detected on scheduled restaging imaging. Total number of lesions irradiated was 57. The most common lesions identified involved more than a single segment of the vertebral column (*n* = 18) which included lesions that spanned the thoracolumbar (*n* = 10), lumbosacral (*n* = 4), and cervicothoracic (*n* = 4) regions. Radiologic cord compression was present in 32% (*n* = 12), epidural extension in 76% (*n* = 28) and compression fracture in 38% (*n* = 14).

**Table 1:** Patient characteristics.

**Characteristics**	**Number**	**Percentage (%)**
Patients	37	
Male	26	70
Female	11	30
**DIAGNOSIS**		
Renal cell cancer (RCC)	9	24
Non-small cell lung cancer (NSCLC)	21	56
Melanoma	7	20
**LUNG/MELANOMA MUTATIONS**		
BRAF	2	5
EGFR	3	8
Others	6	16
**PRESENTING SYMPTOMS**		
Pain	13	35
Asymptomatic	11	30
Weakness (Motor)	2	5
Numbness/Tingling (Sensory)	5	14
Pain and Motor	1	3
Pain and Sensory	5	14
**VERTEBRAL LESIONS**		
Thoracic	16	43
Lumbar	9	24
Cervical	6	16
Sacral	8	17
Multiple levels	18	4
Thoraco-lumbar	10	56
Lumbosacral	4	22
Cervicothoracic	4	22
**RADIOLOGIC FINDINGS**		
Radiologic cord compression	12	32
Epidural extension	28	76
Compression fracture	14	38

Treatment details are shown in Table 2. There were 7 patients (19%) who received both CTLA-4 and PD-1 blockade (2 concurrently, 5 sequentially). Most patients received radiation within 30 days of PD-1 blockade (*n* = 17, 46%), and most started PD-1 inhibitors following the completion of radiotherapy (*n* = 21, 56%). Eleven patients (30%) underwent surgical decompression prior to starting RT.

**Table 2:** Treatment characteristics.

**Characteristics**	**No**.	**Percentage (%)**
**RT IN RELATION TO PD-1 INHIBITION**		
≤ 30 days of immunotherapy	17	46
30-60 days of immunotherapy	7	19
PD-1 inhibitor before RT	9	24
PD-1 inhibitor after RT	21	56
PD-1 inhibitor concurrent with RT	7	19
**RT dose**	8-30 Gy	
Mean	23.5 Gy	
Median	24 Gy	
3 Gy x 10 fractions	11	30
4 Gy x 5 fractions	6	16
4.5 Gy × 5 fractions	5	13
Other regimens	15	41
**RT DELIVERY**		
SRS/SBRT	4	11
Conformal	33	89
Surgical decompression	11	30
**IMMUNOTHERAPY**		
Anti PD-1	37	100
Both anti PD-1 and CTLA-4	7	19

The majority of patients were treated with conformal radiation therapy (*n* = 33, 89%); four patients (11%) received stereotactic body radiotherapy. The median radiation dose delivered was 24 Gy (range 8–30 Gy).

### Outcomes

Table 3 shows the toxicity and response to treatment. Treatment was in general well–tolerated; documented toxicities attributed to radiotherapy included fatigue 16% (*n* = 6), transient pain flare 3% (*n* = 1), nausea/vomiting 3% (*n* = 1) and grade 1 skin changes 3% (*n* = 1). There were no unusual immune related adverse events or immune related adverse events specific to the irradiated site (e.g., colitis in a patient that received lumbar radiation).

**Table 3:** Toxicity and response to treatment.

**Characteristics**	**No**.	**Percentage (%)**
**RADIATION TOXICITY**		
Fatigue	6	16
Nausea/vomiting	1	3
Skin changes (G1/2)	1	3
Pain flare	1	3
**SYMPTOM RELIEF**		
Pain relief	19	100
Motor/sensory improvement	6	46
**RADIOLOGIC RESPONSE**		
CR	3	8
PR	4	10
SD	22	62
PD	4	10
Unknown	4	10

Among patients that received single agent checkpoint inhibition, toxicities developed in 7 of 30 patients (23%) as compared with toxicities in one of 7 patients (14%) who received combined CTLA-4/PD-1 blockade. Five of 17 patients (29%) who received radiation within 30 days of immune checkpoint blockade developed toxicities as compared with two out of 7 patients (28%) who received radiation from 30–60 days of ICB.

Pain control was reported in 42% (*n* = 8/19) of patients at the first follow up after radiation, and eventually in 100% of all patients who presented with pain (15 patients who received single agent checkpoint blockade and 4 patients that received combined CTLA-4 inhibitor therapy). Among the 13 patients with motor or sensory complaints, improvement was noted in 6 (46%). Motor or sensory improvements were more likely in patients who received vertebral radiation after starting PD-1 inhibitors (71vs. 17%, *p* = 0.04). On follow up imaging performed at a median of 30 days (range 7–96 days) from RT, most patients (22 of 33 evaluable patients, 67%) had stability of irradiated lesions, whereas 3 (9%) had a complete local response and 4 (11%) had a partial local response. Four patients had radiographic progression of irradiated lesions; interestingly, these patients also reported some degree of pain relief on subsequent visits following radiotherapy.

None of the patients with single agent checkpoint inhibition showed neurological improvement in terms of sensory and / or motor response (out of 6 patients) compared with 4 of 7 (57%) patients receiving multiagent ICB. Stable disease/treatment response on follow up imaging was seen in all 7 patients who received CTLA-4/PD-1 inhibition as compared to 22 of 30 (73%) patients treated with single agent ICB. Pain control and neurologic improvement were observed in 8 (47%) and 4 (23%) out of 17 patients who received radiation within 30 days of ICB as compared to 5 (71%) and 3 (42%) out of 7 patients who received radiation from 30–60 days of checkpoint inhibition. Disease stability/treatment response was seen in 13 of 17 (76%) patients who received radiation within 30 days from ICB as compared with 6 of 7 patients (85%) who received radiation within 30–60 days of ICB.

**Figure 1:**
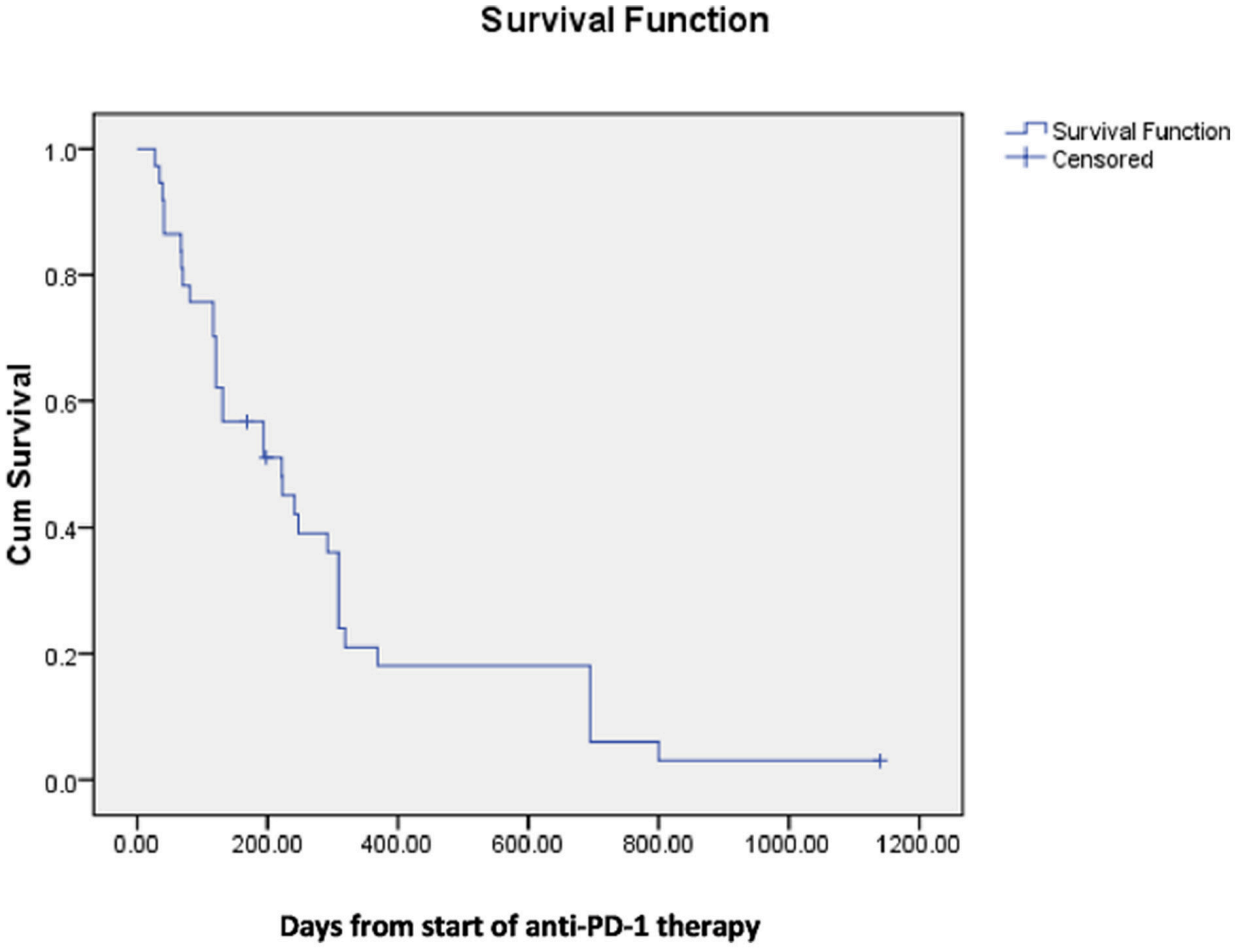
Median survival from start of anti-PD-1 therapy.

**Figure 2:**
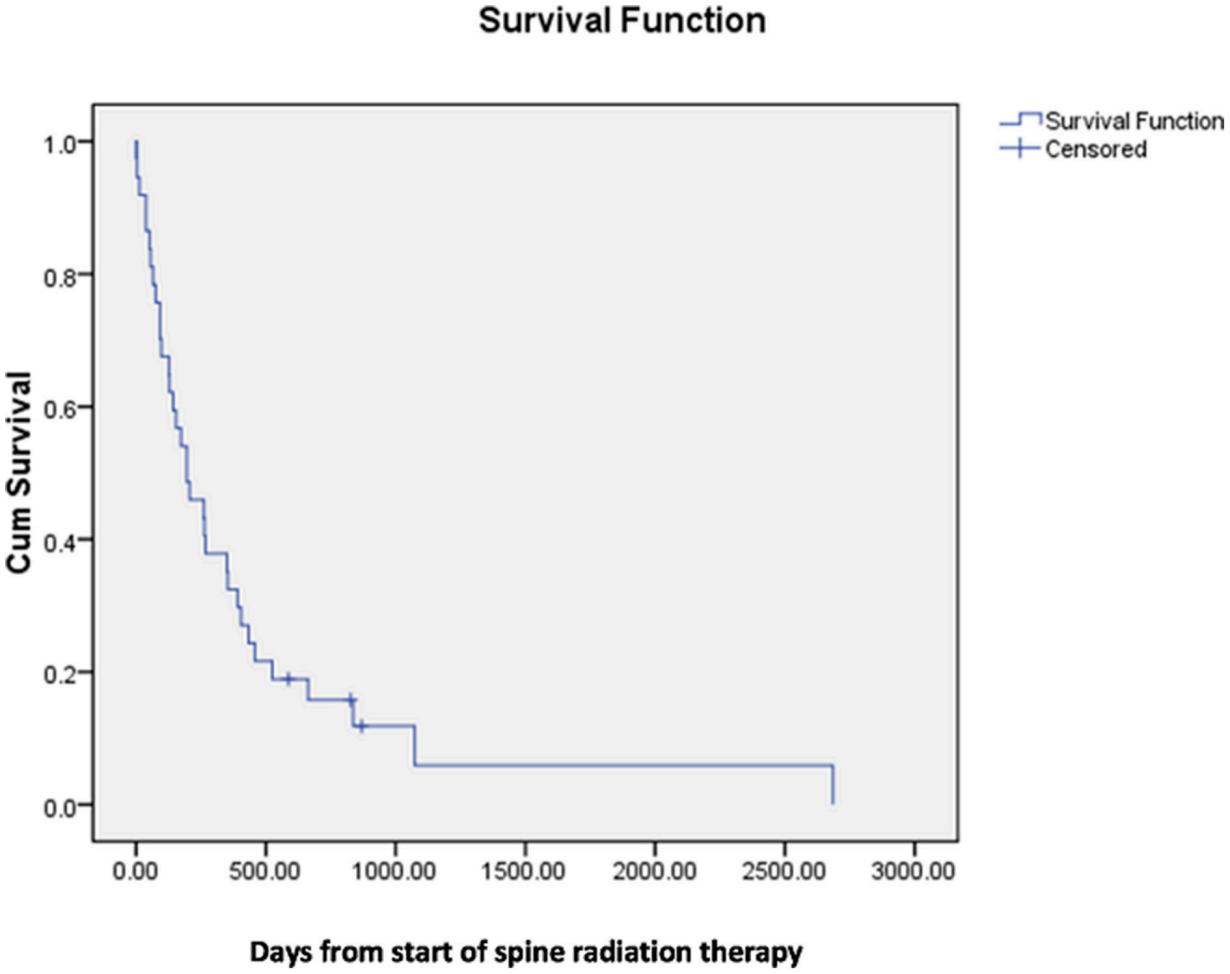
Median survival from start of spinal radiation treatment.

Median survival following spinal radiation was 196 days and median survival from onset of first PD-1 directed therapy was 222 days (Figures 1, 2).

## Discussion

We evaluated 37 patients with metastatic NSCLC, RCC and melanoma treated with immune checkpoint blockade who received radiation therapy directed at 57 lesions in the vertebral column. Reassuringly, treatment was well-tolerated with no unusual immune related adverse events specific to the irradiated site or unexpected toxicities. No patient had transient worsening of motor or sensory symptoms that might be attributed to tumor associated swelling mediated by radiation and /or immune therapy. All patients reported some degree of pain relief on subsequent follow up with numbness or motor improvement additionally seen in 46% of patients who reported these symptoms at baseline. Interestingly, patients were more likely to experience motor/sensory improvement if they were treated with radiation therapy following immune checkpoint blockade.

Radiation therapy is an effective treatment for metastatic cord compression. However, metastases from melanoma, NSCLC, and RCC have been considered more refractory, with variable response rates. For example, a large retrospective study comparing various fractionation schedules for metastatic cord compression found improved motor function following radiation in only 16% of patients with unfavorable histology and 29% of patients experienced deteriorating motor function (16). A prospective study yielded more promising results, with 29% of all patients regaining the ability to walk after radiation, and 66% of unfavorable histology patients either maintaining or regaining the ability to walk after treatment. However, only 56% of all patients experienced pain relief, and both duration of benefit and median survival were relatively short (median 3 months for both) (17). In another study of palliative radiation therapy in the treatment of epidural compression specifically due to metastatic melanoma, complete and partial symptom response was seen in 39 and 46% of sites with a median dose of 28.5 Gy (18).

Although retrospective, our data suggest that rates of symptomatic improvement following vertebral radiation are likely more favorable in patients also treated with PD-1 immune checkpoint inhibitors, particularly in those patients who received immune checkpoint blockade prior to radiation therapy. The observed median survival of almost 7 months is also relatively favorable, with 10 patients (27%) surviving over 1 year, in contrast to the 5% 1 year survival previously observed in unfavorable histology patients (17). The mechanism underlying these favorable results should be investigated further, specifically in regards to the potential synergy between radiotherapy and immune therapy that has been suggested by preclinical and clinical studies (2, 3, 19).

Ideally our results would be validated by prospective studies; however, patients with symptomatic vertebral metastases are a challenging population that are often excluded from systemic therapy trials given the need to initiate radiation on an urgent or emergent basis. In the interim, although follow up is limited, these data provide reassurance not to defer either palliative vertebral radiotherapy or potentially effective systemic immunotherapy in patients who may benefit, including those with vertebral metastases and metastatic cord compression, as well as useful prognostic information with which to help counsel patients. These data are consistent with a growing number of studies that suggest the combination of radiation therapy and PD-1 inhibition are well tolerated (2, 13) as well as with studies that demonstrate improved outcomes and a percentage of long term survivors in a subset of patients with historically poor outcomes, such as patients with brain metastases (20–22).

Future studies should attempt to further optimize the use of palliative vertebral radiation in conjunction with PD-1 checkpoint blockade. Our previous analyses suggested that limited intracranial progression in patients otherwise responding to anti-PD-1 therapy could be adequately addressed by focal radiation, allowing a substantial number of patients to continue on anti-PD-1 therapy for extended periods of time (22). Our numbers were more limited in the current study; however, 7 patients were continued on anti-PD-1 therapy after receiving spine directed radiation. Vertebral radiation could also be effectively delivered prior or concurrent with the start of PD-1 inhibition in patients with high overall burden of disease to reduce tumor mediated immune suppression and delay progression in this sensitive anatomic region, which could allow time for patients to generate effective anti-tumor immune responses that could mediate systemic disease control (9, 10).

## Conclusions

This study shows that palliative radiation therapy administered to vertebral column metastases and spinal cord compression was well tolerated and effective in patients treated with PD-1 directed therapy. There was an encouraging rate of pain reduction and neurological improvement. Additional prospective studies are needed to further evaluate the synergy and efficacy of radiation given in combination with immune checkpoint inhibition for patients with vertebral metastases and spinal cord compression. Given the majority of patients with solid tumors will not respond to immune checkpoint inhibitor monotherapy, developing combination strategies, including those with radiation is critical to improving our ability to harness and direct anti-tumor immune responses.

## Data Availability

The datasets used and/or analyzed during the current study are available from the corresponding author on reasonable request.

## Ethics Statement

This study was retrospective in nature, with DFCI IRB ethics committee approval.

## Author Contributions

JS and MF conceptualized the writing on this topic. MF analyzed and interpreted the patient data regarding the palliative radiation to spine and immune checkpoint inhibition. AB, LP, and MH contributed in writing the manuscript. AT, AS, MA, PO, MK, and TB contributed to the final revision of the manuscript. All authors read and approved the final manuscript.

### Conflict of Interest Statement

AS received honoraria from Bayer Pharmaceuticals, Astellas Pharma, Inc. PO had a research grant from ARMO BioSciences, AstraZeneca/MedImmune, Bristol-Myers Squibb, Merck & Co., Inc., Celldex and was a consultant for Alexion Pharmaceuticals, Amgen, Bristol-Myers Squibb, Celldex, CytomX Therapeutics, Genentech, Neon Therapeutics. TB had a research grant from Templeton Foundation and she is a member of ASCO Palliative Care Steering Committee. JS had a research grant from BMS, Merck., was a consultant for Tilos, was on advisory board for BMS, Debiopharm, AstraZeneca, Nanobiotix, and received travel expenses from BMS; NCI Match Subprotocol Z1D. The remaining authors declare that the research was conducted in the absence of any commercial or financial relationships that could be construed as a potential conflict of interest.

## Abbreviations

PRT: palliative radiation therapy
ICB: immune checkpoint blockade
ECOG: Eastern Cooperative Oncology Group
NSCLC: non-small cell lung cancer
RCC: Renal cell cancer
Gy: Gray
CTCAE: Common Terminology Criteria for Adverse Events
RECIST: Response Evaluation Criteria in Solid Tumors.
